# Frontal adenosine triphosphate markers from ^31^P MRS are associated with cognitive performance in healthy older adults: preliminary findings

**DOI:** 10.3389/fnagi.2023.1180994

**Published:** 2023-08-08

**Authors:** Francesca V. Lopez, Andrew O’Shea, Jens T. Rosenberg, Christiaan Leeuwenburgh, Stephen Anton, Dawn Bowers, Adam J. Woods

**Affiliations:** ^1^Department of Clinical and Health Psychology, College of Public Health and Health Professions, University of Florida, Gainesville, FL, United States; ^2^Center for Cognitive Aging and Memory, McKnight Brain Institute, University of Florida, Gainesville, FL, United States; ^3^Advanced Magnetic Resonance Imaging and Spectroscopy Facility, McKnight Brain Institute, University of Florida, Gainesville, FL, United States; ^4^Department of Aging and Geriatric Research, College of Medicine, University of Florida, Gainesville, FL, United States; ^5^College of Medicine, Institute on Aging, University of Florida, Gainesville, FL, United States; ^6^Department of Neurology, College of Medicine, Norman Fixel Institute for Neurological Diseases, University of Florida, Gainesville, FL, United States

**Keywords:** phosphorous magnetic resonance spectroscopy, adenosine triphosphate, cognition, aging, brain energy metabolism

## Abstract

Aging is associated with declines in mitochondrial efficiency and energy production which directly impacts the availability of adenosine triphosphate (ATP), which contains high energy phosphates critical for a variety of cellular functions. Previous phosphorous magnetic resonance spectroscopy (^31^P MRS) studies demonstrate cerebral ATP declines with age. The purpose of this study was to explore the functional relationships of frontal and posterior ATP levels with cognition in healthy aging. Here, we measured frontal and posterior ATP levels using ^31^P MRS at 3 Tesla (3 T) and assessed cognition using the Montreal Cognitive Assessment (MoCA) in 30 healthy older adults. We found that greater frontal, but not posterior, ATP levels were significantly associated with better MoCA performance. This relationship remained significant after controlling for age, sex, years of education, and brain atrophy. In conclusion, our findings indicate that cognition is related to ATP in the frontal cortex. These preliminary findings may have important implications in the search for non-invasive markers of *in vivo* mitochondrial function and the impact of ATP availability on cognition. Future studies are needed to confirm the functional significance of regional ATP and cognition across the lifespan.

## Introduction

With the number of adults over the age of 65 in the United States expected to double by 2050 (Ortman et al., 2014), cognitive decline and transition to dementia is one of the most prominent public health concerns. Even in the absence of clear neurodegenerative disease, cognition declines in later life which can impact the ability to carry out day-to-day tasks (Burke and Barnes, 2006). Importantly, accumulating evidence indicates changes in brain structure and function are associated with declines in cognition and functional independence (Cabeza, 2002; Raz et al., 2005). Further, cellular changes are a common feature of aging, such as reduced mitochondrial efficiency and energy production (Culmsee and Landshamer, 2006; Grimm et al., 2016; Dienel, 2019). Yet, the relations among reduced mitochondrial function, cognition, and aging remains unclear. Therefore, it is critical to understand how age-related changes in the brain contribute to cognitive aging to identify potential intervention targets to sustain or enhance cognition and functional independence in older adults.

It is well-documented that crystalized cognitive functions (e.g., general knowledge and vocabulary) remain stable, and may even improve, with advancing age (Salthouse, 2010). Conversely, fluid cognitive functions, such as the ability to reason abstractly and solve problems, are sensitive to age-related changes. Such deficits are linked to age-related changes in speed of processing, attention/working memory, and learning efficiency. These changes are particularly prominent in executive functions (Salthouse et al., 2003; Salthouse, 2010). Executive functions refer to a group of higher-order cognitive processes such as updating, inhibition, and set shifting that are critical for carrying out goal-directed behaviors and adapting to novel situations (Miyake et al., 2000; Lezak et al., 2004). Executive functions are heavily dependent on the functional integrity of the prefrontal cortices (Cummings, 1993). Convergently, decreases in brain volume, cortical thickness, and gray matter in the frontal lobes are associated with advancing age, and occur earlier and more severely than any other region of the brain (Raz, 2000; Drag and Bieliauskas, 2010). These structural changes are associated with significant changes in functional activity. Functional magnetic resonance imaging (fMRI) studies show age-related changes in frontal activation patterns (Gazzaley et al., 2004; Reuter-Lorenz et al., 2000). In addition, aging is associated with increased connectivity within frontal regions (Davis et al., 2012), but reduced connectivity with more posterior regions (Dennis et al., 2008). Further, fMRI and PET studies show age-related changes in blood flow across frontal regions are associated with declines on tasks of abstraction, inhibition, switching (Goh et al., 2013), and semantic organization (Nyberg et al., 2010). In addition, declines in mitochondrial function occurs as adults age, which may in part account for age-related structural and functional brain changes (Culmsee and Landshamer, 2006; Leuner et al., 2007; Forester et al., 2010; Grimm et al., 2016).

The Montreal Cognitive Assessment (MoCA) is a brief measure of global cognitive function that is widely used in clinical settings to screen for mild cognitive impairment and dementia (Nasreddine et al., 2005). The MoCA assesses a broad array of cognitive domains including attention/working memory, set-shifting, naming, letter fluency, recent learning and memory, and visuospatial skills. Given that a number of these domains fall under the umbrella of executive functions, the MoCA is considered sensitive to fronto-executive dysfunction. In fact, this tool shows adequate sensitivity to executive dysfunction associated with normal aging, mild cognitive impairment, and other forms of cognitive decline (Hoops et al., 2009; Gluhm et al., 2013). Further, neuroimaging studies demonstrate set-shifting (Zakzanis et al., 2005; Jacobson et al., 2011), letter fluency (Phelps et al., 1997), and attention/working memory (Gerton et al., 2004; Sun et al., 2005) performances on the MoCA are largely associated with brain activity across frontal areas of the brain, though, to a lesser extent, posterior regions are also implicated.

Adenosine triphosphate (ATP) is a high energy phosphate compound found in living cells and supports a variety of cellular functions. In the brain, the majority of ATP is formed within the mitochondria through oxidative phosphorylation. In turn, ATP plays a vital role in neuronal activity and bioenergetics (Bratic and Trifunovic, 2010). Phosphorous magnetic resonance spectroscopy (^31^P MRS) is a non-invasive technique that allows for the *in vivo* investigation of high energy phosphates, such as ATP (Buchli et al., 1994; Ross and Sachdev, 2004; Cady, 2012). Considering the relationship with neuronal activity (Raichle and Mintun, 2006; Bélanger et al., 2011), ATP has been studied in the aging brain. Prior work demonstrates ATP declines with age (Forester et al., 2010; Schmitz et al., 2018). Critically, both whole-brain and frontal ATP have been associated with poor performance on fronto-executive tasks (Volz et al., 1998; Harper et al., 2016). Although promising, the relative functional significance of frontal and posterior ATP on cognition remains unclear.

The purpose of the current preliminary study was to investigate the relationship between frontal and posterior ATP levels with cognitive function in healthy older adults using a widely administered index of cognitive function, the MoCA. The primary ^31^P MRS voxel of interest was placed in the frontal lobes. An additional voxel was placed in the posterior cortex (i.e., parietal lobe) to serve as a control. These selections were made because frontal regions are more strongly related to cognition as assessed by the MoCA than posterior regions (Julayanont and Nasreddine, 2017). The overall hypothesis was that changes in ATP within specific brain regions will be functionally associated with cognition. Specifically, since older adults evidence changes (1) on frontally mediated tasks of executive functions as well as (2) in frontal brain structure-function, and (3) the MoCA carries a high ‘executive’ load, it was predicted that ATP derived from frontal regions, but not posterior regions, would be associated with cognitive performance.

## Materials and methods

### Participants

Data were collected at baseline from participants recruited for the Efficient Brain Study, a Phase II cross-over clinical trial investigating the impact of Fermented Papaya Product (FPP) on brain mitochondrial function, neuroinflammation, and cognitive function in older adults (NCT02771366). The sample included 30 healthy older adults ranging from 65 to 89 years old (see Table 1) recruited at the University of Florida. Inclusion criteria for this study included cognitively normal older adults (i.e., MoCA Total Score ≥ 22; Carson et al., 2018). Study participants could not have a history of head injury or brain damage, severe psychiatric disease or psychological disorder, no formal diagnosis or evidence of dementia, or neurological brain disease. This study was approved by the Western Institutional Review Board (WIRB), and all participants provided written, informed consent. At the baseline visit, participants completed a battery of cognitive assessments, medical history and mood questionnaires, and multimodal MRI/MRS scan. In this study, we used the MoCA and ^31^P MRS data for our analyses.

**TABLE 1:** Study sample characteristics (*n* = 30).

	Mean (SD)	Range
**Demographics**		
Age	73.2 (5.88)	65–89
Education	15.8 (3.24)	12–20
Sex (F/M)	15/15	–
Ethnicity/Race (*n*)		
White	27	–
Black	1	–
Latino	1	–
Other	1	–
**MoCA performance**		
Total score	25.9 (2.25)	23–29
**Tissue fractions**		
Frontal voxel		
Cerebrospinal fluid (CSF) fraction	0.216 (0.052)	0.132–0.338
Gray matter (GM) fraction	0.305 (0.03)	0.230–0.364
White matter (WM) fraction	0.479 (0.04)	0.417–0.570
Posterior voxel		
Cerebrospinal fluid (CSF) fraction	0.200 (0.57)	0.119–0.327
Gray matter (GM) fraction	0.293 (0.03)	0.236–0.353
White matter (WM) fraction	0.506 (0.04)	0.392–0.588
**CSF corrected ratios**		
Frontal voxel		
Adenosine triphosphate (ATP)	0.376 (0.050)	0.282–0.562
Inorganic phosphate (Pi)	0.101 (0.038)	0.033–0.206
Phosphocreatine (PCr)	0.300 (0.043)	0.222–0.387
Phosphomonoesters (PME)	0.140 (0.058)	0.039–0.279
Phosphodiesters (PDE)	0.270 (0.073)	0.117–0.417
Posterior voxel		
Adenosine triphosphate (ATP)	0.362 (0.056)	0.262–0.472
Inorganic phosphate (Pi)	0.119 (0.085)	0.027–0.371
Phosphocreatine (PCr)	0.304 (0.056)	0.183–0.431
Phosphomonoesters (PME)	0.108 (0.076)	0.000–0.355
Phosphodiesters (PDE)	0.282 (0.122)	0.070–0.624

### Montreal Cognitive Assessment (MoCA)

The Montreal Cognitive Assessment (MoCA), a widely used clinical tool to screen for non-normative cognitive decline, was used as an index of global cognition in the current study (Nasreddine et al., 2005). This tool assesses several cognitive domains including attention, concentration, executive functions, memory, language, visuospatial skills, abstraction, calculation, and orientation. Total scores range from 0 to 30, with higher scores reflecting better performance. One point was added to the scores of participants who had 12 years of education or less.

### MRS acquisition and analysis

**FIGURE 1:**
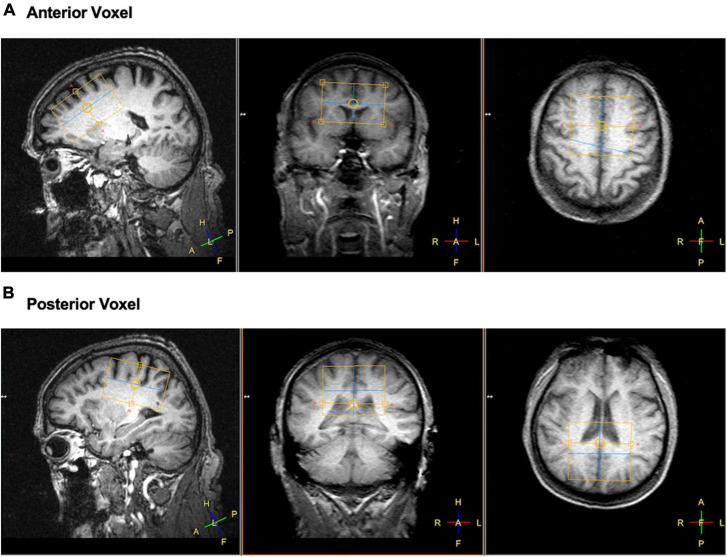
Example of voxel placements for panel **(A)** frontal and **(B)** posterior regions of the brain in left sagittal, coronal, and axial view. Yellow boxes represent the location of the voxel collected.

Neuroimaging data were collected on a Phillips 3 T MRI at the University of Florida McKnight Brain Institute. The scanning session was split into two parts. During the first part, participants were scanned using a dual-tuned (^1^H/^31^P) whole head quadrature coil (Rapid Biomedical, Germany). A T_1_ weighted structural image was acquired (1 mm^3^ isotropic voxel size, 240 × 240 mm acquisition matrix, 160 sagittal slices, Turbo Field Echo) for placement of the two ^31^P MRS voxels. The ^31^P MRS voxels were 156 mL in volume (60 mm a-p direction × 65 mm r-l direction × 40 mm f-h direction). For localized ^31^P MRS acquisition an image-selected *in vivo* spectroscopy (ISIS) sequence was used with adiabatic localization pulses. Parameters for the two voxels were identical and as follows: TR = 4 s; TE = 0.10 ms; 4,096 spectral points; spectral bandwidth = 4500 Hz; 8 phase cycles; and with −100 Hz offset which with 128 averages resulted in 8 min 40 s scan time for each voxel. The voxels were placed at midline to sample from an equal portion of left and right hemisphere and angled to provide sampling of the largest amount of brain tissue while avoiding skull. See Figure 1 for representative voxel placements.

Following ^31^P MRS acquisition, the head coil was switched to a Phillips 32-channel head coil and the participant was repositioned. A second T_1_ 3D MPRAGE weighted structural image was acquired with 1 mm^3^ isotropic voxel size, 240 × 240 mm acquisition matrix, 170 sagittal slices. Parameters for the two voxels were identical and as follows: TE = 3.3 ms, TR = 7.2 ms, TI = 903.51062 ms.

**FIGURE 2:**
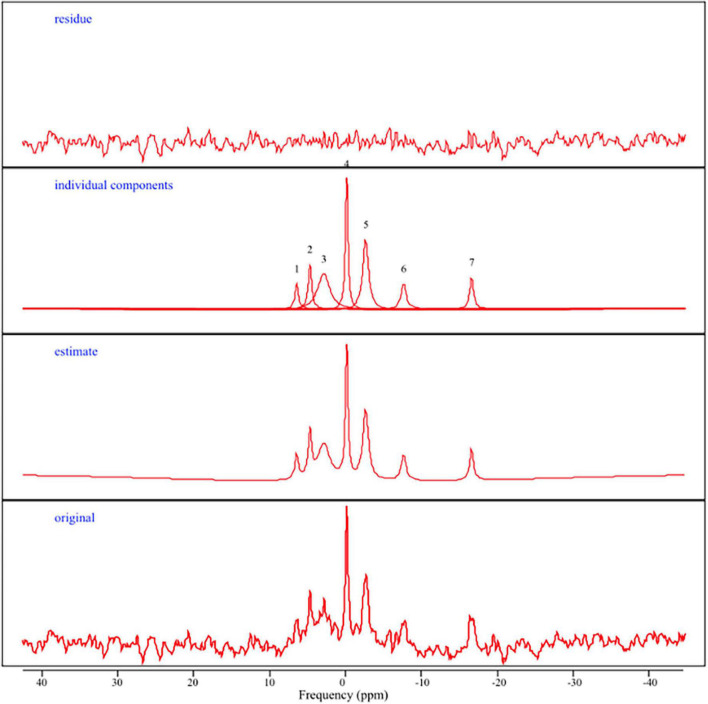
Representative ^31^P MRS spectra. From top to bottom: (residue) the difference between the estimated curve and the original curve; (individual components) representation of ^31^P metabolite peaks including 1 = PME, 2 = Pi, 3 = PDE, 4 = PCr, 5 = gamma ATP, 6 = alpha ATP, 7 = beta ATP; (estimate) spectrum generated after jMRUI/AMARES processing; (original) ^31^P spectrum obtained in the frequency domain in parts per million (ppm).

The AMARES (Advanced Method for Accurate, Robust, and Efficient Spectral Fitting; Vanhamme et al., 1997) time domain spectra fitting algorithm in the jMRUI software package was used to analyze the ^31^P spectra (Stefan et al., 2009). All ^31^P MRS spectra were zero filled with 4,096 points and a 13 Hz Lorentzian line broadening (apodization) was applied before auto-phase shifting to enhance SNR. PCr was adjusted to 0 ppm and constraints for the chemical shifts of the other signals. Seven resonance peaks were manually identified and quantified using previously published estimates (Santos-Díaz and Noseworthy, 2020): gamma, alpha, and beta nucleotide triphosphates (NTP), phosphocreatine (PCr), phosphomonoesters (PME), phosphodiesters (PDE), and inorganic phosphate (Pi). See Figure 2 for a representative output. In general, NTPs provide energy and phosphate group for phosphorylation, and are commonly regarded as ATP in the ^31^P MRS literature (Forester et al., 2010; Ren et al., 2015; Das et al., 2020; Sassani et al., 2020). Thus, in line with prior ^31^P studies, ATP was estimated by calculating a ratio of summed NTP peaks (e.g., alpha-, beta-, and gamma; ATP) to pooled total phosphorous (TP) within a specific region (i.e., frontal and posterior ATP TP ratios).

To control for differing levels of brain atrophy, the acquisition voxels were segmented into gray matter, white matter, and cerebral spinal fluid (CSF) using the software SPM as implemented in the software Gannet. Since we acquired two T_1_ structural scans we used the higher quality 32-channel acquisition for segmentation purposes (after transforming the 32-channel acquisition into the space of the ^31^P acquisition using FSL FLIRT; Zhang et al., 2001; Smith et al., 2004). All regression models were run using CSF fraction or gray and white matter as a covariate. All models significant with the CSF fraction approach were also significant for the gray and white matter approach. Therefore, CSF fraction was used as a covariate in subsequent analyses because it makes fewer tissue-specific assumptions and is consistent with previously published approaches in ^31^P MRS (Rijpma et al., 2018; Schmitz et al., 2018).

### Data analysis

First, a multiple linear regression was performed to examine the relationship between demographic variables (age, education, and sex) and MoCA scores (cognitive performance). Next, separate multiple regressions were performed to examine the relationship between age, CSF fraction, and ATP TP ratios (frontal, posterior). Finally, separate multiple linear regressions were performed to examine the relationship between ATP TP ratios (frontal, posterior) and MoCA scores (overall cognitive performance), controlling for age, education, sex, and CSF fraction. These analyses were repeated in exploratory analyses with supplemental ^31^P metabolites including Pi, PCr, PME, and PDE. All statistical analyses were conducted using the Statistical Package for the Social Sciences (SPSS) Version 25.

## Results

### MoCA scores as a function of demographic variables

The relationship between MoCA scores and demographic variables were investigated using multiple linear regression. The results of this regression indicated the demographic variables did not account for a significant proportion of variance in MoCA performance (R^2^ = 0.015, *F*(3, 29) = 1.15, *p* = 0.349). No significant predictors emerged.

### ^31^P Metabolites as a function of age

The relationship between frontal ATP TP ratios and age was examined using multiple linear regression, while controlling for CSF fraction. The overall model was significant (R^2^ = 0.14, *F*(2, 29) = 3.37, *p* = 0.049). At the trending level, CSF fraction was positively related to age (β = 0.42, *p* = 0.058), while frontal ATP TP ratios were not (β = 0.05, *p* = 0.825). Exploratory analyses indicated that there was not a relationship between age and supplemental ^31^P metabolites in the frontal voxel (all *p*’s > 0.150).

A similar multiple linear regression analysis examined the relationship between posterior ATP TP ratios and age, while controlling for CSF fraction. The overall model was significant (R^2^ = 0.22, *F*(2, 29) = 5.16, *p* = 0.013). CSF fraction was positively related to age (β = 0.47, *p* = 0.009). Posterior ATP ratios were not related to age (β = 0.20, *p* = 0.238). Exploratory analyses indicated that there was not a relationship between age and supplemental ^31^P metabolites in the posterior voxel (all *p*’s > 0.327).

### MoCA scores as a function of ^31^P metabolites

**FIGURE 3:**
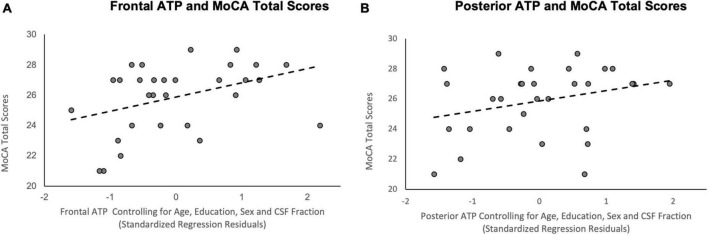
Scatterplots of the relationship between ATP TP ratios and MoCA total scores. **(A)** Frontal ATP TP ratios; **(B)** Posterior ATP TP ratios; *R*^2^ reflects variance explained from the partial correlation between regional ATP TP ratios and MoCA total scores; *X*-axis = standardized residual of ATP TP ratios after controlling for age, sex, education, and CSF fraction; *Y*-axis = MoCA total scores.

The relationship between frontal ATP TP ratios and MoCA scores were examined using multiple linear regression, while controlling for sex, age, education, and CSF fraction. The results of this regression indicated ATP in this region accounted for a significant amount of the variance in MoCA scores (R^2^ = 0.41, *F*(5, 24) = 3.38, *p* = 0.020; See Figure 3A). Higher frontal ATP TP ratios were related to better cognitive performance on the MoCA (β = 0.49, *p* = 0.021), while controlling for CSF fraction (β = −0.68, *p* = 0.003). Conversely, age (β = 0.05, *p* = 0.81), sex (β = 0.22, *p* = 0.21), and education (β = 0.14, *p* = 0.41) were not related to MoCA scores. In contrast, exploratory analyses indicated that there was not a relationship between MoCA scores and supplemental ^31^P metabolites in the frontal voxel (all *p*’s > 0.338).

A similar multiple linear regression analysis examined the relationship between posterior ATP TP ratios and MoCA scores. The results of this regression demonstrated that the five predictors accounted for a significant amount of variance in MoCA scores (R^2^ = 0.38, *F*(5, 24) = 2.91, *p* = 0.034; See Figure 3B). Among the variables, CSF fraction (β = −0.50, *p* = 0.023) and, to a lesser extent, sex (β = 0.38, *p* = 0.049), were significantly related to MoCA scores. Posterior ATP TP ratios did not account for a significant amount of variance in MoCA performance (β = 0.32, *p* = 0.91), nor did age (β = 0.14, *p* = 0.54) or years of education (β = 0.29, *p* = 0.14). Exploratory analyses showed a similar non-significant relationship between MoCA scores and supplemental ^31^P metabolites in the posterior voxel (all *p*’s > 0.194).

## Discussion

Aging is associated with declines in cognition and mitochondrial function. However, the relationship between mitochondrial function and cognition remains unclear. The purpose of this preliminary study was to explore the functional relationships of frontal and posterior ATP on cognition in a sample of healthy older adults. We had two preliminary findings. First, we did not find a significant relationship between ^31^P metabolites and age, which is not consistent with previous studies (Ross and Sachdev, 2004; Forester et al., 2010; Schmitz et al., 2018). A potential explanation is range restriction due to a small sample size suppressed a possible effect. Future studies are needed to determine whether the relationship between ^31^P metabolites and age is robust in more demographically heterogenous and larger samples.

Our second major finding was that we found frontal, but not posterior, ATP was associated with MoCA total scores. Specifically, higher availability of frontal ATP was associated with better performance on the MoCA. This relationship persisted after controlling for demographic characteristics (i.e., age, sex, and education) and age-related atrophy (i.e., CSF voxel). However, other frontal ^31^P metabolites were not related to MoCA performance. These findings are partially consistent with previous ^31^P MRS research, which has demonstrated associations between ATP (i.e., frontal or whole brain) and cognition (i.e., executive functions) (Volz et al., 1998; Harper et al., 2016). We expanded on this previous research in several ways. In terms of methodology, we examined ATP in two regions of the brain within the same sample. Most importantly, we demonstrated the differential relationship between regional ATP and cognition in older adults. This pattern of results is consistent with the “last in, first out” hypothesis, which suggests brain regions last to develop show increased vulnerability to the aging process (Raz, 2001). Specifically, there are preferential volumetric and functional changes in the frontal lobes relative to other regions of the brain in aging (Gazzaley et al., 2004; Dennis et al., 2008; Davis et al., 2012; Reuter-Lorenz et al., 2000), which correspond to declines in executive functions (Drag and Bieliauskas, 2010). Speculating beyond the results of this study, ^31^P MRS markers of ATP within a focal brain region may map onto the functional role of that region in mediating cognition. An alternative explanation for our findings is that other factors impacted this observed relationship, despite our efforts to control for potential covariates. For example, aging is accompanied by changes in metabolic and/or cardiovascular health, (e.g., glycemic control, hypertension), which have been shown to have negative effects on the brain and cognition (Suji and Sivakami, 2004; Tzourio et al., 2014). Together, this may raise the question as to whether metabolic and/or cardiovascular health may attenuate or exacerbate the relationship between ATP and cognition. Future work may benefit from examining the relationship between metabolic and cardiovascular risk on ATP, and its resulting impact on cognition.

In the broader context, plasticity of mitochondria is compromised in aging, which directly disrupts homeostatic regulation of the fusion-fission cycle that allows mitochondrial functional and genetic complementation, and the proper distribution of newly synthesized mitochondria during cell division. This is further compounded by altered mitochondrial biogenesis and degradation (Berman et al., 2008). In turn, there is an accumulation of damaged or dysfunctional mitochondria in cells, which are then rendered unable to execute vital cellular functions, such as production of ATP (Bratic and Trifunovic, 2010). Our findings demonstrate that ATP is associated with behavior, and specifically cognition, in a sample of healthy older adults. Clinically relevant, dysfunctional or damaged mitochondria are implicated in pathological aging processes, including Alzheimer’s disease (AD) (Bertoni-Freddari et al., 2004). As with normal aging, the use of ^31^P MRS to investigate brain-behavior relationships and progression of clinical symptoms has been vastly underutilized (Ross and Sachdev, 2004). Additional investigation is warranted to determine the clinical utility of regional ATP as marker of preclinical AD risk in healthy older adults. Such an approach may elucidate potential mitochondrial mechanisms contributing to normal and pathological aging processes.

There are limitations to this study. First, our sample included a small population (*n* = 30) that was mostly white and well-educated participants who were cognitively normal. While our findings make a relevant contribution to our understanding of the aging brain, they should also be viewed as preliminary due to limits in overall sample size and the findings should be confirmed in a larger sample with a less homogeneous demographic population. Second, our results are limited to cognition as indexed by a global cognitive screener. Even so, the MoCA is a widely used clinical tool that has adequate psychometric properties and sensitivity to cognitive changes in several clinical populations as well as aging. Therefore, learning whether there is a relationship between ^31^P metabolites and MoCA total scores is highly clinically relevant. Future studies are needed to confirm the functional significance of regional ATP and cognition. Third, the current study used a cross-sectional design. Thus, the longitudinal course of these observed relationships is unknown. Future research should explore the stability of these relationships and its relationship to the progression of cognitive decline over time.

In conclusion, this study adds to the growing body of literature examining the associations between ^31^P metabolites and cognition in older adults. Importantly, we provide evidence that frontal, but not posterior, ATP is associated with global cognition, even after controlling for well-known predictors of cognition including age, education, sex, and brain atrophy. These findings may have important implications in the search for non-invasive markers of *in vivo* mitochondrial function and the impact of ATP on behavior across the lifespan.

## Data availability statement

The raw data supporting the conclusions of this article will be made available by the authors, without undue reservation.

## Ethics statement

The studies involving human participants were reviewed and approved by the Western Institutional Review Board. The patients/participants provided their written informed consent to participate in this study.

## Author contributions

All authors made substantial contributions to the development of the study, analysis and/or interpretation of data, drafting of the manuscript, and/or providing critical review of the manuscript, and approved the final version.
